# Supplementary material for: SV40 exploits the Nesprin-2-SUN1-KPNA4 axis for stepwise targeting and entry into the host nucleus to promote infection
Source: PLoS Pathog. 2026 Jun 11;22(6):e1014344. doi: 10.1371/journal.ppat.1014344 (PMC13278577; doi:10.1371/journal.ppat.1014344)
Supplement: Attachment — Submitted filename: Gohmann and Tsai (Rebuttal letter).docx [file ppat.1014344.s002.docx]

We thank the reviewers for their insightful comments. Because Reviewers #1 and #2 had no major comments, we focused the revision on addressing comments from Reviewer #3 by performing additional experiments and changing the text.

**Reviewer #3**

**Comment 1:** A central conceptual issue is how SUN1, an inner nuclear membrane protein, influences the capture of cytosolic SV40 via Nesprin-2 on the outer nuclear membrane. The data in Fig. 3G suggest that SUN1 is required for the Nesprin-2–SV40 interaction, yet the mechanistic basis for this is not resolved. Given that SV40 accumulates at ER foci in SUN1-depleted cells, it is critical to determine whether microtubule-dependent transport toward the nucleus is impaired. Specifically does SUN1 depletion disrupt BICD2–dynein–Nesprin-2 interactions? Is directional movement of SV40 toward the nuclear envelope affected?

As requested, we performed an additional Nesprin-2 immunoprecipitation experiment to investigate its interaction with the BicD2-dynein motor complex. Specifically, we found that depletion of SUN1 decreased the interaction between Nesprin-2 and a dynein component (dynein intermediate chain) (**Fig. 3J**). Additionally, our data in Figures 2A/B revealed that SV40 spatially separated from the nuclear membrane under SUN1 depletion (when compared to Scr and SUN2 KD), indicating that directional trafficking of SV40 towards the nuclear membrane is disrupted. Together, we believe these results support the model that SUN1 promotes Nesprin-2-dependent capture of the dynein motor complex to facilitate SV40 ER-nuclear membrane transport.

**Comment 2:** The finding that SUN1 is required for Nesprin-2 binding to SV40 is interesting and intriguing and raises an important question. Does SUN1 directly participate in viral recruitment, or does it regulate the conformation/organization of Nesprin-2 at the NPC? Although the authors did not observe any visual difference in Nesperin -2 localization in Sun1 depleted cells in Fig 1, it is important to determine if the observed effects are indirect via motor recruitment.

As requested, we performed an additional Nesprin-2 immunoprecipitation experiment to determine whether SUN1 regulates the conformation of Nesprin-2 (localized on the outer nuclear membrane). Specifically, we transfected a previously validated FLAG-tagged “mini” Nesprin-2 (or control FLAG-2xmNeonGreen) and found that depletion of SUN1 reduced endogenous Nesprin-2’s interaction with mini-Nesprin-2 (**Fig. 3K**), indicating impaired oligomerization of Nesprin-2 under SUN1 KD. This finding suggests that SUN1 may function to promote a high order oligomer complex of Nesprin-2 proteins. However, as we state in the text, the precise mechanism by which SUN1 (or SUN2) regulates Nesprin-2 conformation/activity is a critical question in the field that remains to be fully resolved.

**Comment 3**: Whether SV40 is preferentially targeted to specific NPC-associated regions, or whether KPNA4 recruitment occurs at defined nuclear envelope subdomains is unclear from the proposed models and the data. At minimum, this limitation should be more clearly discussed.

As requested, text discussing this limitation was added to the Discussion section.

**Comment 4:** The specificity of KPNA4 (but not KPNA1/2) in promoting SV40 infection is a key finding. However, the mechanistic basis for this specificity is not explored for a article that needs to be published in PLOS Pathogens. For instance the NLS in VP2/3, does that play any role in this Nesperin-2/Sun1/KPNA4 mediated nuclear targeting. There are different classes of NLS in proteins and each NLS can perform distinct function and associate with distinct at nuclear import factors. The authors need to carefully examine the role of VP NLS in this context or at the least discuss this.

As requested, we investigated the reliance of the shared C-terminal NLS of VP2/3 on KPNA4-mediated trafficking of SV40 into the nucleus. We performed an additional KPNA4 co-immunoprecipitation experiment with transfected VP2 or VP2ΔNLS and found that only VP2 (but not VP2ΔNLS) was pulled down (**Fig. 6I**), indicating that the NLS in VP2 and VP3 is a critical motif for interactions with KPNA4.

To further investigate this interaction, we incubated CV-1 cell extracts with biotinylated peptides containing amino acid sequences of the shared NLS between VP2/3 (VP2/3 NLS) or the shared NLS with lysine and arginine mutated to alanine (VP2/3 NLS K/R🡪A). After precipitation of the peptides with Streptavidin beads, the pulled down materials were subjected to SDS-PAGE and immunoblotting using an antibody against KPNA4. Importantly, we found that only wildtype VP2/3 NLS, but not VP2/3 NLS K/R🡪A (or a Steptavidin beads only control) precipitated KPNA4 (**Fig. 6J**). These findings indicate that the shared NLS of SV40 VP2/3 is not only required but also sufficient to support KPNA4 binding.

**Comment 5**: The quantification of disassembled versus assembled virus in Fig. 2E is not sufficiently clear. It would be important to show VP1 levels across individual gradient fractions rather than only the combined quantification presented. From the western blots, the distribution of VP1 across fractions does not appear to differ substantially between control and SUN1 KD conditions, raising questions about how disassembly is being assessed. In addition, given that BICD2 is implicated in SV40 disassembly and transport, it is unclear whether SUN1 depletion affects BICD2 function or engagement with the virus. If disassembly is indeed unchanged, this would suggest that SUN1 acts downstream of BICD2-mediated disassembly.

As requested, we re-quantified the VP1 level in each fraction in Fig. 2E. A 2-way repeated measures ANOVA found no significant difference in VP1 distribution across fractions between control cells and SUN1 KD cells. This new quantification is now shown in the right graph of Fig. 2E.

Additionally, as reviewer #3 states, the BicD2 interaction with the virus should remain unchanged in SUN1 KD cells if disassembly is occurring. To test this, we performed a FLAG-BicD2 immunoprecipitation experiment and found that its engagement with SV40 VP1 is unchanged between control Scr cells and SUN1 KD cells (**Fig. 2F**). Collectively, these data demonstrate that SV40 binds to BicD2 and experiences cytosolic disassembly in SUN1-depleted cells.

**Comment 6:** Preventing SV40 from accessing the nuclear interior during infection and halting them to stay exposed in the cytosol, does that trigger an innate response.

We agree that this is an interesting and important question. However, we hope this reviewer agrees that this question is likely beyond the scope of this present investigation and would be better addressed by a follow-up study.

**Minor Comments**

**Reviewer #3**

**Comment 1:** Line 80 is a bit confusing, what is extracted out, is it SV40 or the genetic material? Or are the authors talking about naked virus getting into the cytoplasm

We have edited the text to make it clear that the entire SV40 virion is extracted from the ER to the cytoplasm.

**Comment 2:** Typo error in line 115

Thank you. We have corrected this typo.

**Comment 3:** The authors may have to look carefully at Fig 1F, the cells shown seem to be Myco positive (from DAPI stain).

We thank the reviewer for their concern. We regularly test our cells for mycoplasma contamination via PCR-based protocols (PMID: 37216121). We now used this protocol and found no detection of mycoplasma contamination in our CV-1 cells (see below). We also note that the extranuclear DAPI signal the reviewer observes is only present in figures with transfected DNA, and not in images without transfection (compare Figures 1F, 4B, 6E to Figures 1J, 2A, 2B, 5E, 6G). This indicates that the plasmid DNA is likely being detected by the DAPI counterstain, which has been previously tested and observed (PMID: 35058555).

**Comment 4:** The orientation in which figures are oriented is a bit confusing in this article. Examples are Figure 3D which is not in alignment with others in the same panel and another if Fig 5A. In Fig 5A, it might be better to show Kd data of siRNA first and then T-Ag levels.

We thank the reviewer for their suggestions. However, we prefer to maintain the orientation we have presented the figures in.

**Comment 5:** In fig 6D, consider putting the graph next to the western (Fig 6C) or changing the Y axis to include Sun1 Flag IP

As requested, we have included SUN1-3xFLAG into the y-axis for Figure 3D.

**Reviewer #1**

**Comment 1:** The only minor critique is that some of the representative microscopy data, as in Fig. 1F, was quite pixelated, and greater care should be taken to export high quality images in the appropriate format for review

We thank the reviewer for this suggestion and have included images with higher resolution in Figures 1J, 2A, 2B, 4B, 5E, 6E, and 6G.

**Additional notes**

Please note that we updated our model figure (Fig. 6K) to be more descriptive and detailed.
