# Supplementary material for: SV40 exploits the Nesprin-2-SUN1-KPNA4 axis for stepwise targeting and entry into the host nucleus to promote infection
Source: PLoS Pathog. 2026 Jun 11;22(6):e1014344. doi: 10.1371/journal.ppat.1014344 (PMC13278577; doi:10.1371/journal.ppat.1014344)
Supplement: Peer review history

# Peer review: Decision Letter 0

**DOI:** 10.1371/journal.ppat.1014344.r001

**Authors:** Alison McBride

2 Apr 2026

-->PPATHOGENS-D-26-00573

SV40 exploits the Nesprin-2-SUN1-KPNA4 axis for stepwise targeting and entry into the host nucleus to promote infection

PLOS Pathogens

Dear Dr. Tsai,

Thank you for submitting your manuscript to PLOS Pathogens. After careful consideration, we feel that it has merit but does not fully meet PLOS Pathogens's publication criteria as it currently stands. Therefore, we invite you to submit a revised version of the manuscript that addresses the points raised during the review process.

Please submit your revised manuscript by Jun 01 2026 11:59PM. If you will need more time than this to complete your revisions, please reply to this message or contact the journal office at plospathogens@plos.org. When you're ready to submit your revision, log on to https://www.editorialmanager.com/ppathogens/ and select the 'Submissions Needing Revision' folder to locate your manuscript file.

Please include the following items when submitting your revised manuscript:

* A letter that responds to each point raised by the editor and reviewer(s). You should upload this letter as a separate file labeled 'Response to Reviewers'. This file does not need to include responses to any formatting updates and technical items listed in the 'Journal Requirements' section below.

* A marked-up copy of your manuscript that highlights changes made to the original version. You should upload this as a separate file labeled 'Revised Manuscript with Track Changes'.

* An unmarked version of your revised paper without tracked changes. You should upload this as a separate file labeled 'Manuscript'.

If you would like to make changes to your financial disclosure, competing interests statement, or data availability statement, please make these updates within the submission form at the time of resubmission. Guidelines for resubmitting your figure files are available below the reviewer comments at the end of this letter.

We look forward to receiving your revised manuscript.

Kind regards,

Walter J. Atwood

Academic Editor

PLOS Pathogens

Alison McBride

Section Editor

PLOS Pathogens

Sumita Bhaduri-McIntosh

Editor-in-Chief

PLOS Pathogens

orcid.org/0000-0003-2946-9497

Michael Malim

Editor-in-Chief

PLOS Pathogens

orcid.org/0000-0002-7699-2064

**Journal Requirements:**

1) Please ensure that the CRediT author contributions listed for every co-author are completed accurately and in full.

At this stage, the following Authors/Authors require contributions: Luke Gohmann, and Billy Tsai. Please ensure that the full contributions of each author are acknowledged in the "Add/Edit/Remove Authors" section of our submission form.

The list of CRediT author contributions may be found here: https://journals.plos.org/plospathogens/s/authorship#loc-author-contributions

2) We ask that a manuscript source file is provided at Revision. Please upload your manuscript file as a .doc, .docx, .rtf or .tex. If you are providing a .tex file, please upload it under the item type u2018LaTeX Source Fileu2019 and leave your .pdf version as the item type u2018Manuscriptu2019.

**Reviewers' Comments:**

Reviewer's Responses to Questions

**Part I - Summary**

Please use this section to discuss strengths/weaknesses of study, novelty/significance, general execution and scholarship.

Reviewer #1: The stepwise penetration of SV40 particles through ER membranes, partial disassembly in the cytoplasm, and trafficking to nuclear pores provides an elegant entry mechanism that remains only partially understood. The Tsai laboratory has long been a leader in this field, advancing understanding of this complex entry mechanism. Here, they provide new mechanistic insights, implicating the SUN-KASH complex proteins Nesprin2 and SUN1 in mediating transport of SV40 particles from the cytosolic face of the ER to the nuclear periphery, where KPNA4 is involved in targeting particles to nuclear pores to complete the uncoating process.

This manuscript is exceptionally well written and the data is very clearly and logically presented. Methods are well described.

The authors’ claims are very well supported by the dataset. These findings represent a significant advance in our understanding of the process of polyomavirus entry into host cells. Undoubtedly, some of these findings may be relevant to understanding how other viruses traffic to the nucleus as well.

The data analysis and presentation is of exceptionally high quality.

Reviewer #2: This is strong well designed set of experiments demonstrating step-by-step how Nesprin-2-SUN1-KPNA4 targets and assists the nuclear entry of SV40 into the nucleus. I really cannot not think of anything that needs to changed or added.

Reviewer #3: This manuscript investigates how SV40 exploits components of the LINC complex and nuclear import machinery to achieve nuclear entry. The authors identify a stepwise mechanism in which the Nesprin-2–SUN1 complex mediates targeting of cytosolic virus to the nuclear envelope, followed by KPNA4-dependent translocation through the nuclear pore. The study addresses an important question in DNA virus biology and presents several interesting observations, particularly the specificity for SUN1 over SUN2 and for KPNA4 among importin-α isoforms.

However, while the data generally supports the requirement of Nesperin-2 /Sun1 for SV40 nuclear targeting, several key mechanistic aspects remain insufficiently resolved. In particular, the relationship between SUN1 (inner nuclear membrane), Nesprin-2 (outer nuclear membrane), cytosolic trafficking, and NPC engagement are not fully clarified. A careful examination of these would further strengthen the manuscript and at this stage requires major revision.

**********

**Part II – Major Issues: Key Experiments Required for Acceptance**

Please use this section to detail the key new experiments or modifications of existing experiments that should be absolutely required to validate study conclusions.

Generally, there should be no more than 3 such required experiments or major modifications for a "Major Revision" recommendation. If more than 3 experiments are necessary to validate the study conclusions, then you are encouraged to recommend "Reject".

Reviewer #1: (No Response)

Reviewer #2: N/A

Reviewer #3: 1) A central conceptual issue is how SUN1, an inner nuclear membrane protein, influences the capture of cytosolic SV40 via Nesprin-2 on the outer nuclear membrane. The data in Fig. 3G suggest that SUN1 is required for the Nesprin-2–SV40 interaction, yet the mechanistic basis for this is not resolved. Given that SV40 accumulates at ER foci in SUN1-depleted cells, it is critical to determine whether microtubule-dependent transport toward the nucleus is impaired. Specifically does SUN1 depletion disrupt BICD2–dynein–Nesprin-2 interactions? Is directional movement of SV40 toward the nuclear envelope affected?

2) The finding that SUN1 is required for Nesprin-2 binding to SV40 is interesting and intriguing and raises an important question. Does SUN1 directly participate in viral recruitment, or does it regulate the conformation/organization of Nesprin-2 at the NPC? Although the authors did not observe any visual difference in Nesperin -2 localization in Sun1 depleted cells in Fig 1, it is important to determine if the observed effects are indirect via motor recruitment.

3) Whether SV40 is preferentially targeted to specific NPC-associated regions, or whether KPNA4 recruitment occurs at defined nuclear envelope subdomains is unclear from the proposed models and the data. At minimum, this limitation should be more clearly discussed.

4) The specificity of KPNA4 (but not KPNA1/2) in promoting SV40 infection is a key finding. However, the mechanistic basis for this specificity is not explored for a article that needs to be published in PLOS Pathogens. For instance the NLS in VP2/3, does that play any role in this Nesperin-2/Sun1/KPNA4 mediated nuclear targeting. There are different classes of NLS in proteins and each NLS can perform distinct function and associate with distinct at nuclear import factors. The authors need to carefully examine the role of VP NLS in this context or at the least discuss this.

5) The quantification of disassembled versus assembled virus in Fig. 2E is not sufficiently clear. It would be important to show VP1 levels across individual gradient fractions rather than only the combined quantification presented. From the western blots, the distribution of VP1 across fractions does not appear to differ substantially between control and SUN1 KD conditions, raising questions about how disassembly is being assessed. In addition, given that BICD2 is implicated in SV40 disassembly and transport, it is unclear whether SUN1 depletion affects BICD2 function or engagement with the virus. If disassembly is indeed unchanged, this would suggest that SUN1 acts downstream of BICD2-mediated disassembly.

6) Preventing SV40 from accessing the nuclear interior during infection and halting them to stay exposed in the cytosol, does that trigger an innate response?

**********

**Part III – Minor Issues: Editorial and Data Presentation Modifications**

Please use this section for editorial suggestions as well as relatively minor modifications of existing data that would enhance clarity.

Reviewer #1: The only minor critique is that some of the representative microscopy data, as in Fig. 1F, was quite pixelated, and greater care should be taken to export high quality images in the appropriate format for review.

Reviewer #2: N/A

Reviewer #3: 1) Line 80 is a bit confusing, what is extracted out, is it SV40 or the genetic material? Or are the authors talking about naked virus getting into the cytoplasm.

2) Typo error in line 115

3) The authors may have to look carefully at Fig 1F, the cells shown seem to be Myco positive (from DAPI stain).

4) The orientation in which figures are oriented is a bit confusing in this article. Examples are Figure 3D which is not in alignment with others in the same panel and another if Fig 5A. In Fig 5A, it might be better to show Kd data of siRNA first and then T-Ag levels.

5) In fig 6D, consider putting the graph next to the western (Fig 6C) or changing the Y axis to include Sun1 Flag IP

**********

PLOS authors have the option to publish the peer review history of their article (what does this mean?). If published, this will include your full peer review and any attached files.

If you choose “no”, your identity will remain anonymous but your review may still be made public.

**Do you want your identity to be public for this peer review?**  For information about this choice, including consent withdrawal, please see our Privacy Policy.

Reviewer #1: No

Reviewer #2: No

Reviewer #3: No

[NOTE: If reviewer comments were submitted as an attachment file, they will be attached to this email and accessible via the submission site. Please log into your account, locate the manuscript record, and check for the action link "View Attachments". If this link does not appear, there are no attachment files.]

**Figure resubmission:**

-->While revising your submission, we strongly recommend that you use PLOS’s NAAS tool (https://ngplosjournals.pagemajik.ai/artanalysis) to test your figure files. NAAS can convert your figure files to the TIFF file type and meet basic requirements (such as print size, resolution), or provide you with a report on issues that do not meet our requirements and that NAAS cannot fix.

After uploading your figures to PLOS’s NAAS tool - https://ngplosjournals.pagemajik.ai/artanalysis, NAAS will process the files provided and display the results in the "Uploaded Files" section of the page as the processing is complete. If the uploaded figures meet our requirements (or NAAS is able to fix the files to meet our requirements), the figure will be marked as "fixed" above. If NAAS is unable to fix the files, a red "failed" label will appear above. When NAAS has confirmed that the figure files meet our requirements, please download the file via the download option, and include these NAAS processed figure files when submitting your revised manuscript.

**Reproducibility:**

To enhance the reproducibility of your results, we recommend that authors of applicable studies deposit laboratory protocols in protocols.io, where a protocol can be assigned its own identifier (DOI) such that it can be cited independently in the future. Additionally, PLOS ONE offers an option to publish peer-reviewed clinical study protocols. Read more information on sharing protocols at https://plos.org/protocols?utm_medium=editorial-email&utm_source=authorletters&utm_campaign=protocols

# Author comment: Author response to Decision Letter 1

**DOI:** 10.1371/journal.ppat.1014344.r002

1 Jun 2026

Attachment Submitted filename: Gohmann and Tsai (Rebuttal letter).docx

# Editor report: Decision Letter 1

**DOI:** 10.1371/journal.ppat.1014344.r003

**Authors:** Alison McBride

3 Jun 2026

Dear Prof. Tsai,

We are pleased to inform you that your manuscript 'SV40 exploits the Nesprin-2-SUN1-KPNA4 axis for stepwise targeting and entry into the host nucleus to promote infection' has been provisionally accepted for publication in PLOS Pathogens.

Before your manuscript can be formally accepted you will need to complete some formatting changes, which you will receive in a follow up email. A member of our team will be in touch with a set of requests.

Please note that your manuscript will not be scheduled for publication until you have made the required changes, so a swift response is appreciated.

IMPORTANT: The editorial review process is now complete. PLOS will only permit corrections to spelling, formatting or significant scientific errors from this point onwards. Requests for major changes, or any which affect the scientific understanding of your work, will cause delays to the publication date of your manuscript.

Should you, your institution's press office or the journal office choose to press release your paper, you will automatically be opted out of early publication. We ask that you notify us now if you or your institution is planning to press release the article. All press must be co-ordinated with PLOS.

Thank you again for supporting Open Access publishing; we are looking forward to publishing your work in PLOS Pathogens.

Best regards,

Walter J. Atwood

Academic Editor

PLOS Pathogens

Alison McBride

Section Editor

PLOS Pathogens

Sumita Bhaduri-McIntosh

Editor-in-Chief

PLOS Pathogens

orcid.org/0000-0003-2946-9497

Michael Malim

Editor-in-Chief

PLOS Pathogens

orcid.org/0000-0002-7699-2064

***********************************************************

Reviewer Comments (if any, and for reference):

# Editor report: Acceptance letter

**DOI:** 10.1371/journal.ppat.1014344.r004

**Authors:** Alison McBride

Dear Prof. Tsai,

We are delighted to inform you that your manuscript, "SV40 exploits the Nesprin-2-SUN1-KPNA4 axis for stepwise targeting and entry into the host nucleus to promote infection," has been formally accepted for publication in PLOS Pathogens.

We have now passed your article onto the PLOS Production Department who will complete the rest of the pre-publication process. All authors will receive a confirmation email upon publication.

The corresponding author will soon be receiving a typeset proof for review, to ensure errors have not been introduced during production. Please review the PDF proof of your manuscript carefully, as this is the last chance to correct any scientific or type-setting errors. Please note that major changes, or those which affect the scientific understanding of the work, will likely cause delays to the publication date of your manuscript. Note: Proofs for Front Matter articles (Pearls, Reviews, Opinions, etc...) are generated on a different schedule and may not be made available as quickly.

Soon after your final files are uploaded, the early version of your manuscript, if you opted to have an early version of your article, will be published online. The date of the early version will be your article's publication date. The final article will be published to the same URL, and all versions of the paper will be accessible to readers.

For Research Articles, you will receive an invoice from PLOS for your publication fee after your manuscript has reached the completed accept phase. If you receive an email requesting payment before acceptance or for any other service, this may be a phishing scheme. Learn how to identify phishing emails and protect your accounts at https://explore.plos.org/phishing.

Thank you again for supporting open-access publishing; we are looking forward to publishing your work in PLOS Pathogens.

Best regards,

Sumita Bhaduri-McIntosh

Editor-in-Chief

PLOS Pathogens

orcid.org/0000-0003-2946-9497

Michael Malim

Editor-in-Chief

PLOS Pathogens

orcid.org/0000-0002-7699-2064
